# Purification, Characterization and Bioactivity of a New Homodimeric Lectin From *Vicia Altissima* (*Fabaceae*) Seeds

**DOI:** 10.1002/pei3.70047

**Published:** 2025-04-02

**Authors:** Youssef Elamine, Julio Girón‐Calle, Manuel Alaiz, Javier Vioque

**Affiliations:** ^1^ Food Phytochemistry Department Instituto de la Grasa (C.S.I.C.), Campus Universidad Pablo de Olavide Sevilla Spain

**Keywords:** antiproliferative activity, mannose, section *Pedunculatae*, subgenus *Vicilla*, THP‐1 cells

## Abstract

*Vicia altissima* Desf. (Fabaceae) belongs to subgenus *Vicilla*, section *Pedunculatae*. It is a perennial herb that grows in wet ravines with dense vegetation in western Mediterranean countries. The only population that exists in Spain is under critical threat of extinction. Although lectins are abundant in the seeds from several *Vicias* belonging to subgenus *Vicilla*, the presence of lectins in section *Pedunculatae* has not been investigated. Purification of lectins from *V. altissima* seeds was carried out by albumin extraction according to solubility in water and gel filtration chromatography using a Superose 12 column. SDS‐PAGE and native PAGE analyses revealed single bands at 38 and 87 kDa, respectively, indicating that this protein is a homodimer. The lectin exhibited a high affinity for mannose and glucose and inhibited the proliferation of THP‐1 cells. Seed lectins from *Vicia* species belonging to sect. *Cracca* in subg. *Vicilla* are, in general, more sensitive to inhibition by N‐acetylgalactosamine than to inhibition by glucose or mannose. On the contrary, the seed lectin from *V. altissima* , belonging to sect. *Pedunculatae*, has a higher affinity for mannose and glucose than for N‐acetylgalactosamine. Our results show the presence of a lectin with antiproliferative activity in the seeds from *V. altissima* , indicating that this lectin has potential health‐promoting and diagnostic applications. These potential applications could have a positive effect on the preservation of this wild legume, which is represented in Spain by only one endangered population.

## 1 Introduction

**FIGURE 1:**
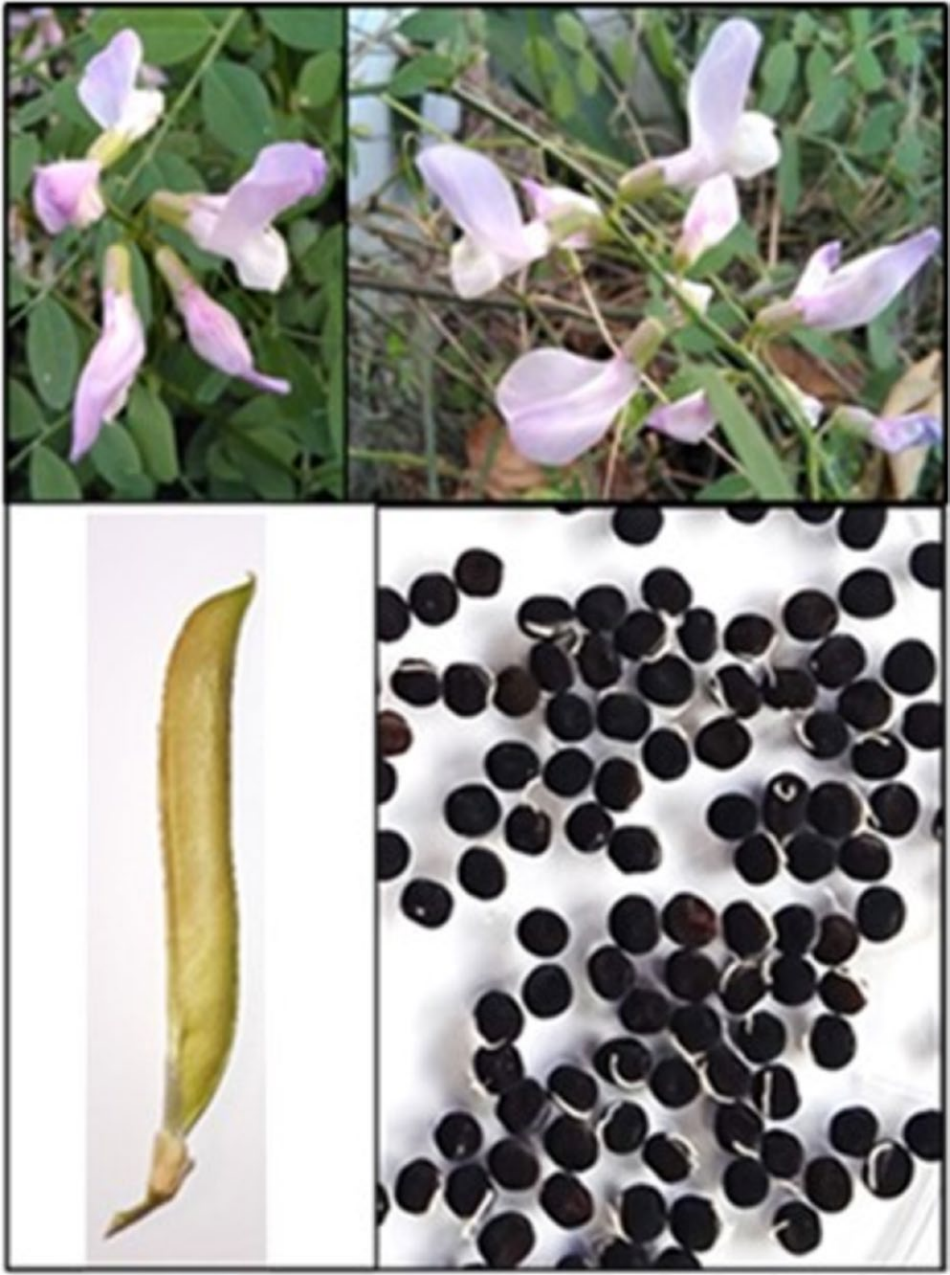
Flowers (up left and right pictures), fruit (bottom left picture) and seeds (bottom right picture) from *V. altissima* .

The *Vicia* genus belongs to the *Fabaceae* family and includes more than 200 species that are native to Eurasia, America, and Africa, although they are especially abundant in the Northern Hemisphere (Hanelt and Mettin 1989). *Vicia* includes subgenus *Vicia* and *Vicilla*. The former is a complex taxonomic group that includes 17 sections (Kupicha 1976) that have been established according to the anatomy of the vascular system, stipules, epidermis, inflorescences, flowers, fruits, and seeds. *V. altissima* , *V. onobrychioides*, and *V. cedretorum* belong to section *Pedunculatae*, which is characterized by amphistomatic leaves, linear leaflets, stenonychioid vexillum, and pubescent style dorsally compressed. This section is considered a natural monophyletic group within the *Vicia* genus and is present in Mediterranean countries (Jaaska 2005). *V. altissima* is found in Spain, France, Croatia, Italy, and Algeria. *V. onobrychioides* is more widely distributed and is found in Algeria, Albania, Bulgaria, France, Greece, Switzerland, Spain, Italy, former Yugoslavia, Portugal, Morocco, and Tunisia. *V. cedretorum* is endemic to Morocco. *V. altissima* grows in wet ravines with dense riparian vegetation. It is a perennial, hermaphrodite, entomophilous herb with rhizomes, and uses ballochory (ballistic) seed dispersal (Figure 1).

Although biochemical studies dealing with *Vicia* species in sect. *Pedunculatae* are scarce, total (Pastor‐Cavada et al. 2011) and free (Megías et al. 2016) amino acid compositions of the seeds from some species belonging to this section have been reported. The protein content, amino acid composition, and nutritional characteristics of the seed protein in this section were similar to those reported for other *Vicias*. Despite being deficient in tryptophan, the amino acid composition of *V. altissima* seeds is the most balanced according to FAO recommendations (FAO 1985). Unlike other species in subgenus *Vicilla*, such as those belonging to sect. *Cracca*, seeds in sect. *Pedunculatae* do not contain the non‐proteic amino acid canavanine (Megías et al. 2016), which is one of the antinutritional compounds that some *Vicias* accumulate in their seeds as protection against predators. Antinutritional chemicals in legumes, in general, include low molecular weight molecules such as vicine, convicine, cyanogenic glucosides, and non‐proteic amino acids, as well as proteins such as protease inhibitors and lectins (Muzquiz et al. 2012). Lectins are non‐enzymatic proteins that bind carbohydrates and are abundant in the seeds of many legumes (Lagarda‐Diaz et al. 2017). They have a defensive role against predation because they bind to carbohydrates in the brush border of enterocytes, causing undesirable effects including nutrient malabsorption (Vasconcelos and Oliveira 2004). Lectins are very abundant in cotyledons and also represent a source of nitrogen during germination (Rüdiger and Gabius 2001). In addition to their physiological roles in plants, lectins are considered potential health‐promoting components due to their antiproliferative activity on a variety of cancerous cell lines (Mazalovska and Kouokam 2018; Ribeiro et al. 2018), as exemplified by the lectins from *Astragalus membranaceus* (Bai et al. 2018), *Erophaca baetica* (Megías et al. 2013), and *Canavalia ensiformis* (Kim et al. 2008). This antiproliferative effect of lectins may be mediated by induction of apoptosis. For example, cell death induced by treatment with concanavalin A in melanoma A375 and hepatocellular carcinoma HepG2 cells was due to activation of the mitochondrial apoptotic pathway (Chang and Lei 2008). Lectins in legumes are divided into two groups according to their quaternary structure. Thus, the single‐chain lectins are characterized by having subunits of similar molecular weight, while the subunits in the so‐called two‐chain lectins differ in molecular weight (Rüdiger and Gabius 2001).

*Vicia* genus belongs to Tribe *Vicieae*, which also includes genera *Pisum*, *Lens*, *Lathyrus*, and *Vavilovia* (Kupicha 1981). Seed lectins have been purified from all of these genera except for genus *Vavilovia*, and most of them exhibit affinity for glucose and mannose (Cavada et al. 2021). However, lectins from *V. villosa* and *V. palaestina* have a higher affinity for N‐acetylgalactosamine and the Tn antigen (Kaladas et al. 1981; Elamine et al. 2021). Lectins from Tribe *Vicieae* exhibit a variety of functional properties, including antiproliferative, antiviral, and antimicrobial effects (Kabir et al. 2013; Uematsu et al. 2012; El‐Araby et al. 2020), and have also been used to determine sperm viability (Mendoza et al. 1992) and in the construction of microarrays to analyze cancer glycomics (Fry et al. 2011). The purification of lectins from the seeds of several *Vicias* belonging to sect. *Cracca*, subgenus *Vicilla*, has been recently described (Megías et al. 2019; Elamine et al. 2021). These are single‐chain lectins and have a high affinity for N‐acetyl‐galactosamine. In addition, it has been reported that the lectin from *V. palaestina* , belonging to the same section, inhibits the proliferation of THP‐1 cells. The objective of the present work was to determine whether *V. altissima* , belonging to sect. *Pedunculatae*, is a source of seed lectins with biochemical and antiproliferative properties similar to those reported for *Vicias* from sect. *Cracca*.

## 2 Experimental

### 2.1 Materials

Fetal bovine serum, tissue culture media, antibiotics, trypsin–EDTA solution, and non‐essential amino acids for cell culture were purchased from Invitrogen/Gibco (Barcelona, Spain). Diethyl ethoxy‐methylene‐malonate, D‐L‐α‐aminobutyric acid, bromophenol blue, Coomassie brilliant blue G, and red blood cells were from Sigma Aldrich. All other reagents were of analytical grade. *V. altissima* seeds were collected from the only known population at the Iberian Peninsula, which is located at Barranco de Tremecen, Sierra de Cabrera, Turre, Almería, Spain, in July 2007. Seeds were stored at –20°C and used to grow new plants at the Instituto de la Grasa (C.S.I.C.) greenhouse facilities during the years 2018 and 2019. Voucher specimens of *V. altissima* are deposited at the Herbarium of the Department of Plant Biology and Ecology, University of Seville (SEV‐240697).

### 2.2 Purification of *V. altissima* Lectin

The *V. altissima* lectin was purified from pH 4.0 protein extracts by ultrafiltration followed by size exclusion chromatography using a Superose 12 gel filtration column coupled to an FPLC AKTA Purifier System, as previously described (Elamine et al. 2021).

### 2.3 Amino Acid Analysis

Pure lectin was hydrolyzed at 110°C in 6 N HCl for 20 h. Amino acids were analyzed by RP‐HPLC after derivatization with diethyl ethoxymethylenemalonate according to Alaiz et al. (1992). Tryptophan was determined by RP‐HPLC after alkaline hydrolysis, as previously described (Yust et al. 2004).

### 2.4 SDS‐Page

SDS‐PAGE was performed according to Schägger and von Jagow (1987) and Elamine et al. (2021).

### 2.5 Native PAGE

Native PAGE was carried out using Mini‐PROTEAN TGX (4%–20%) precast gels from BIO‐RAD (CA, USA) as previously described (Elamine et al. 2021).

### 2.6 Erythrocytes Agglutination Assay

Agglutinating activity was assayed using fixed trypsinized erythrocytes as previously described (Elamine et al. 2021). Agglutinating activity was categorized as negative or positive by visual inspection. The lectin from *Canavalia ensiformis* (1 μg/well) was used as a positive control, and incubations of erythrocytes with no lectin were used as a negative control.

### 2.7 THP‐1 Cell Culture and Proliferation Assay

THP‐1 monocytic cells were cultured and exposed to *V. altissima* lectin in a humidified 5% CO_2_ incubator. Cells were seeded in 96‐well microplates (10^4^ cells/well) and cultured for 2, 3, and 4 days after the addition of lectin. Proliferation of THP‐1 cells was measured using the 3‐(4,5‐dimethylthiazol‐2‐yl)2,5‐ diphenyltetrazolium bromide (MTT) assay adapted from Mosmann (1983) as previously described (Elamine et al. 2021).

### 2.8 Statistical Analysis

Principal component analysis (PCA) was performed using the MATLAB 2018a software. The amino acid composition data that was used for cluster and principal component analysis was obtained from the following sources. The amino acid composition of the lectins from *Canavalia ensiformis* (ID 2098436), *Glycine max* (ID 3891966), *Phaseolus lunatus* (ID 8920387), *Dioclea grandiflora* (ID 5107577), *Robinia pseudoacacia* (ID 538529), *Vigna unguiculata* (ID 388103), and *Wisteria floribunda* (ID 1064245663) were calculated from their protein sequences. The amino acid composition of lectins from *Vicia faba* , *Pisum sativum* , *Lathyrus sativus* , *Lathyrus cicera* , and *Lens culinaris* corresponds to unpublished results. Finally, the amino acid composition of the lectins from some *Vicia* species has been previously published (Megías et al. 2019; Elamine et al. 2021).

## 3 Results

### 3.1 Purification of *V. altissima* Seed Lectin

**FIGURE 2:**
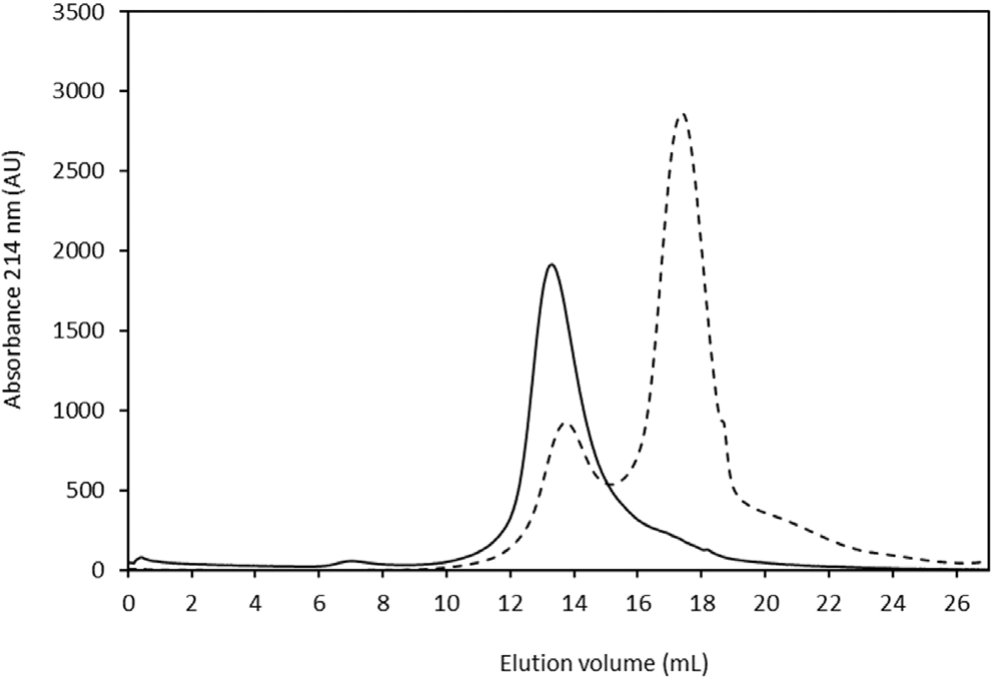
FPLC‐Superose 12 gel filtration chromatography of a *V. altissima* seed water extract (dotted line) and purified seed lectin (solid line).

**FIGURE 3:**
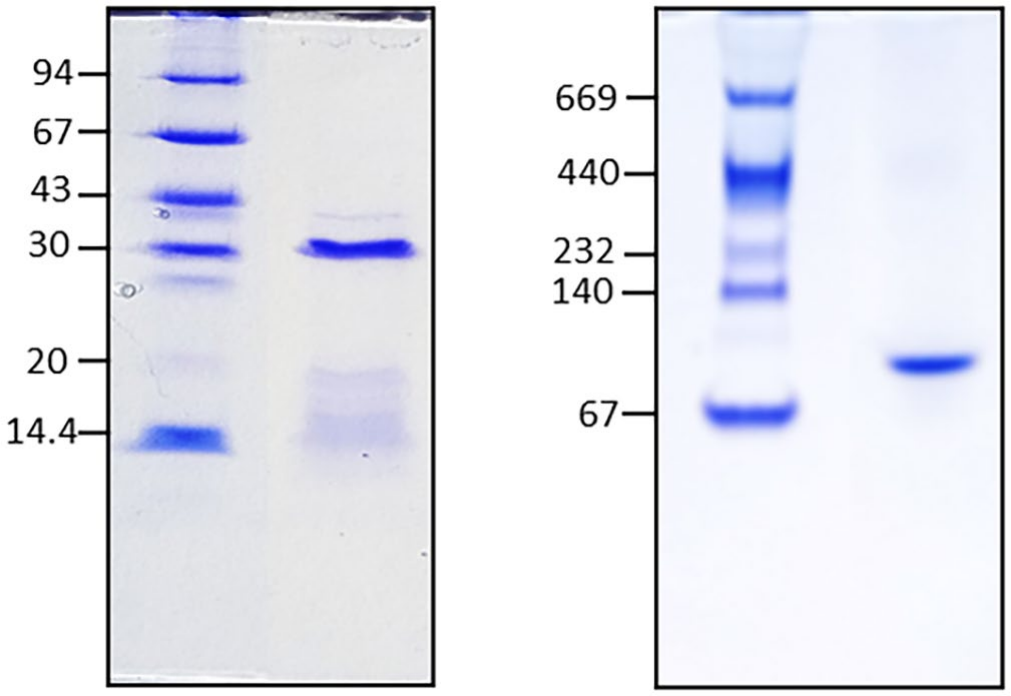
SDS‐PAGE (left) and native‐PAGE (right) of the purified *V. altissima* seed lectin. Molecular weight standards are shown on the side (kDa).

Purification of lectins from *V. altissima* seeds started with the classic separation of albumins and globulins according to solubility in water (Osborne 1908). Globulins are storage proteins that represent the most abundant protein fraction in leguminous seeds and precipitate at a certain pH called the isoelectric point. Albumins, including enzymes, protease inhibitors, and lectins, remain soluble at the isoelectric pH and incorporate most functional proteins. Extraction of *V. altissima* flour at the isoelectric point, pH 4 (Vioque et al. 2012, 2020), yielded an extract containing albumins, as well as low molecular weight secondary components such as polyphenols and free amino acids, that were partially removed by ultrafiltration using a 5 kDa membrane. The resulting fraction was then applied to a Superose 12 FPLC gel filtration column. As shown in Figure 2 (dotted line), this fraction still contained a good deal of low molecular weight components that elute after albumins at 18 mL. The eluted fractions were assayed for red blood cells agglutinating activity and pooled accordingly, and were reapplied to the column until the lectin fraction eluted as a single peak (Figure 2, solid line). Electrophoretic analysis of this fraction by SDS‐PAGE revealed a single band at 38 kDa, while native PAGE, which preserves quaternary structure, yielded a single band at 87 kDa (Figure 3). According to the nomenclature suggested by Van Damme et al. (2008), the lectin purified from *V. altissima* seeds should be named VICALTA. This name is derived from the first three letters of the generic name (*Vicia*) followed by the first three letters of the specific name (*altissima*), followed by an “a”

### 3.2 Erythrocyte Agglutinating Activity

**FIGURE 4:**
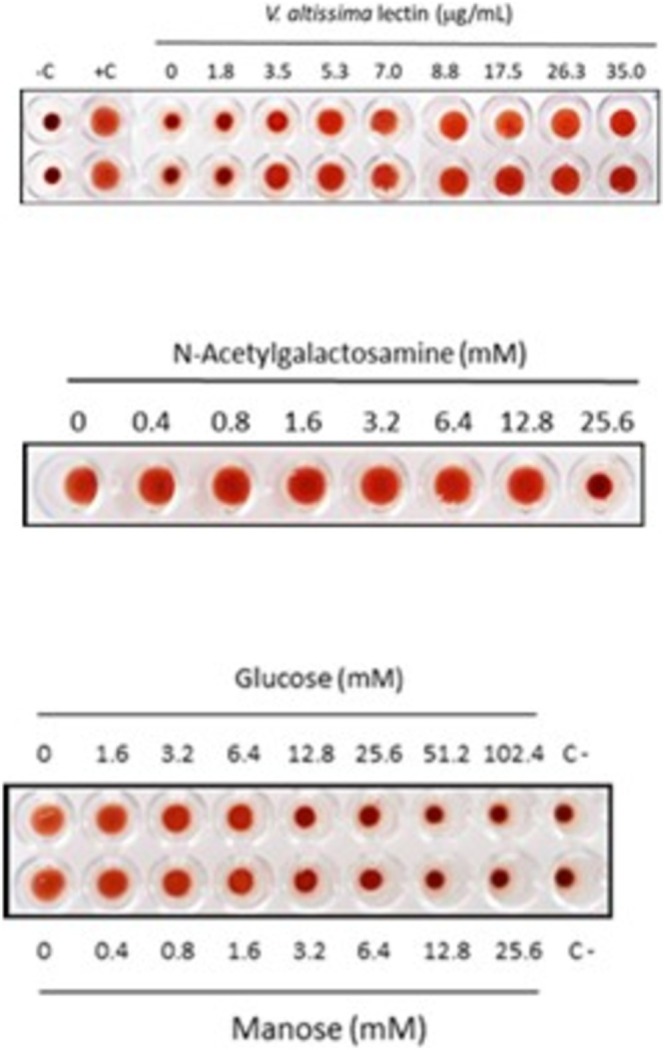
Agglutination of red blood cells by the lectin purified from *V. altissima* seeds (upper panel). Inhibition of the agglutinating activity by N‐Acetylgalactosamine (middle panel), glucose and mannose (lower panel).

Lectins are characterized by being able to bind to sugar residues on the cell surface, causing agglutination of red blood cells (Sharon and Lis 1972). As shown in Figure 4 (upper panel), the lowest concentration of lectin from *V. altissima* seeds that caused full agglutination of red blood cells was 5.3 μg lectin/mL. The agglutination of erythrocytes by lectins is inhibited by sugars that bind to the active site of lectins and compete with the sugar residues on the cell surface, including glucose, mannose, and N‐acetylgalactosamine (Jiang et al. 2015). Some lectins are inhibited by several different sugars, while others are strongly inhibited by one specific sugar. The seed lectin from *V. altissima* has a higher affinity for mannose and glucose than for N‐acetylgalactosamine (Figure 4, lower panels).

### 3.3 Amino Acid Composition and Principal Components Analysis (PCA)

**FIGURE 5:**
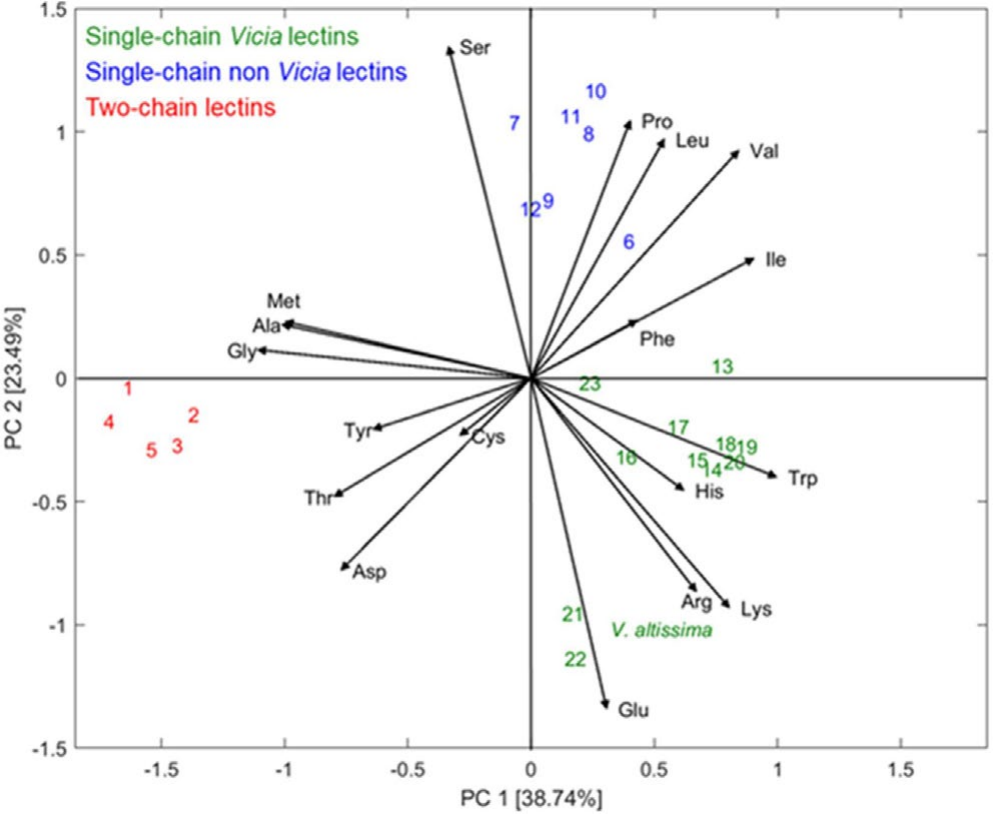
Principal Component Analysis of different one‐chain and two‐chain legume lectins and *V. altissima* lectin. *Lathyrus sativus* (1), *Pisum sativum* (2), *Lens culinaris* (3), *Vicia faba* (4), *Lathyrus cicera* (5), *Wisteria floribunda* (6), *Vigna unguiculata* (7), *Robinia pseudoacacia* (8), *Dioclea grandiflora* (9), *Glycine max* (10), *Phaseolus lunatus* (11), *Canavalia ensiformis* (12), *Vicia benghalensis* (13), *Vicia dasycarpa* (14), *Vicia monantha* (15), *Vicia villosa* (16), *Vicia cracca* (17), *Vicia vicioides* (18), *Vicia pseudocracca* (19), *Vicia disperma* (20), *Vicia monardii* (21), *Vicia tenuifolia* (22), *Vicia palaestina* (23).

The amino acid composition of the lectin purified from *V. altissima* was determined (Table 1) and compared to the amino acid composition of lectins from related legumes by principal component analysis (PCA) in order to search for phylogenetic and lectin‐dependent relations (Figure 5). The first principal component (PC1) represents the direction along which the data have the most variance (38.74%). The second principal component (PC2), perpendicular to PC1, indicates the second highest amount of variance in the data. Hence, PC1 and PC2 explain the maximum variance in the data and accounted for 62.2% of the variance. Single‐chain lectins (blue and green numbers) are clearly discriminated from two‐chain lectins (red numbers) by the first principal component. In addition, the second principal component discriminates between single‐chain *Vicia* lectins (green numbers) and single‐chain non‐*Vicia* lectins (blue numbers). The amino acids Glu, Arg, Lys, His, and Trp predominate in *Vicia* single‐chain lectins, while Ser, Pro, Leu, Val, Phe, and Ile are the most abundant in the single‐chain lectins not belonging to *Vicia* (not shown). Considering taxonomic classification, species belonging to the tribe *Fabeae* (*Lathyrus*, *Lens*, *Pisum* and *Vicia*) are characterized by negative PC2 values, while species outside of this tribe exhibit positive PC2 values and are consequently located to the right in the diagram.

**TABLE 1:** Amino acid composition of the lectin purified from *V. altissima* seeds.

Amino acids	Mean ± SD
Aspartic acid^a^	13.94 ± 0.03
Glutamic acid^b^	14.97 ± 0.04
Serine	6.04 ± 0.00
Histidine	2.11 ± 0.03
Glycine	5.35 ± 0.01
Threonine	7.77 ± 0.04
Arginine	4.25 ± 0.04
Alanine	7.04 ± 0.01
Proline	2.85 ± 0.00
Tyrosine	2.66 ± 0.01
Valine	4.80 ± 0.01
Methionine	0.40 ± 0.01
Cysteine	0.54 ± 0.06
Isoleucine	4.55 ± 0.02
Tryptophan	2.53 ± 0.05
Leucine	5.61 ± 0.03
Phenylalanine	4.53 ± 0.01
Lysine	10.06 ± 0.02

*Note:* Data (g amino acids/100 g lectin) are the average of two independent determinations ± standard deviation.

^a^ Aspartic acid + asparagine.

^b^ Glutamic acid + glutamine.

### 3.4 Antiproliferative Activity of *V. altissima* Lectin

**FIGURE 6:**
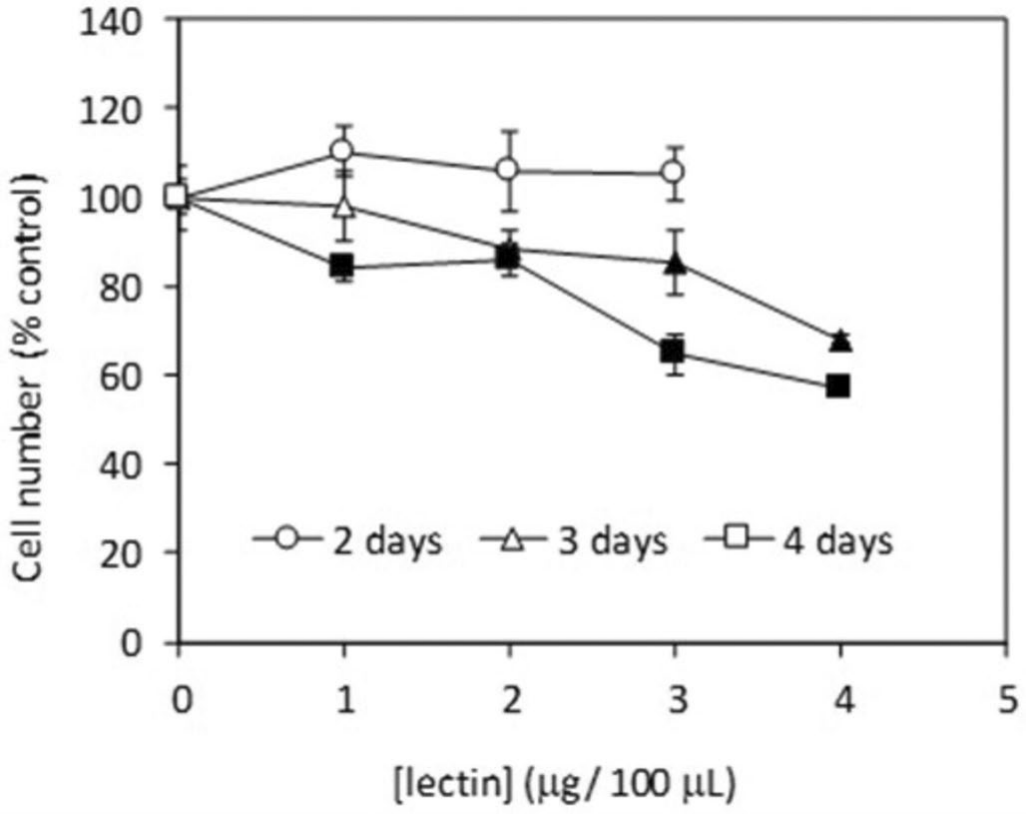
Effect of *V. altissima* lectin on proliferation of THP‐1 cells. Cells (10^4^ cells/well) were cultured in 96‐well plates in culture medium containing 10, 20, 30, or 40 μg lectin/mL. Cell number was estimated after incubation for 2 (circles), 3 (triangles) and 4 (squares) days using the MTT assay. Data represent average ± standard deviation of six incubations. Solid markers indicate significant differences (*p* < 0.05) as compared to control.

THP‐1 cells, a monocytic cell line derived from a leukemia case, were exposed to the lectin purified from *V. altissima* in order to determine its potential effect on the proliferation of cancerous cells. Cells were exposed to 10, 20, 30, and 40 μg lectin/mL for up to 4 days, resulting in significant inhibition of proliferation after treatment for 3 and 4 days (Figure 6).

## 4 Discussion

A lectin was purified from the seeds of *V. altissima* seeds by gel filtration chromatography of an albumin protein extract. SDS‐PAGE analysis showing only one protein band indicates that it belongs to the single‐chain legume lectin group. Its molecular weight is similar to that previously reported for lectins in subgenus *Vicilla* sect. *Cracca* (Megías et al. 2019; Elamine et al. 2021). This observation, in addition to the apparent molecular weight of the protein in non‐denaturing conditions, as revealed by native PAGE, indicates that the lectin purified from *V. altissima* is a homodimer made up of two identical subunits. Another homodimeric lectin with a similar apparent molecular weight, as revealed by native and SDS‐PAGE, has been purified from *V. tetrasperma* (Devi et al. 2009), which also belongs to subgenus *Vicilla*.

The red blood cell agglutinating activity of the lectin was similar to those reported for lectins purified from other *Vicia* species belonging to subg. *Vicilla*, including *V. monantha* (5.6 μg/mL), *V. vicioides* (5.6 μg/mL) and *V. benghalensis* (6.0 μg/mL) (Megías et al. 2019). The agglutinating activity of the lectins from *Vicia* species belonging to sect. *Cracca* in subg. *Vicilla* is, in general, more sensitive to inhibition by N‐acetylgalactosamine than to inhibition by glucose or mannose (Megías et al. 2019). On the contrary, the lectin from *V. altissima* , belonging to sect. *Pedunculatae*, has a higher affinity for mannose and glucose than for N‐acetylgalactosamine (Figure 4, lower panels). Mannose‐binding lectins are widely distributed in legumes, including species from taxonomically distant tribes such as *Vicieae*, *Diocleae*, and *Hedysareae*. It is believed that these lectins play a role in the recognition of glycans from microorganisms and plant predators that are rich in mannose (dos Santos Silva et al. 2019). Few amino acids in the active site of the lectin are responsible for specific sugar recognition by establishing hydrogen bonds with the latter. It has been suggested that the lack of interaction of these amino acids with a specific oxygen atom in mannose and glucose, which presents a different spatial conformation, may explain the specificity for these sugars (Barre et al. 2001), although the lectin from *V. altissima* exhibited higher specificity for mannose than for glucose. Mannose/glucose lectins have also been described in *Vicieae* species, including *Lens culinaris* , *Pisum sativum* , and also in *Vicias* such as *V. cracca* , *V. sativa* , and *V. faba* (Cavada et al. 2021). Several of these lectins in the tribe *Vicieae* have also shown antiproliferative activity (Cavada et al. 2021).

Comparison of the amino acid composition of this lectin with the amino acid composition of lectins from related taxa by principal component analysis revealed interesting groupings consistent with both taxonomy and quaternary structure. Thus, single‐chain lectins were clearly differentiated from dimeric lectins, and single‐chain lectins from *Vicia* were in addition discriminated from single‐chain lectins not belonging to this genus. Nevertheless, PCA did not position the lectin from *V. altissima* close to other mannose/glucose lectins such as the lectins from *Canavalia* and *Dioclea* and those in tribe *Vicieae*, as mentioned above. This is not a conflicting result because the overall amino acid composition does not necessarily reflect the very minor differences in the amino acid sequence that determine affinity for specific sugar residues. Thus, changes in just two amino acid residues in the sugar‐binding site may determine the carbohydrate‐binding specificity (Loris et al. 1998). Lectins bind to specific sugars and glycans, including those in glycoproteins and glycolipids on the cell surface. Malignant cells display a specific array of sugars on their surface, which allows for lectins to discriminate between malignant and normal cells. This specificity is being used to target tumor cells, and lectins from plants are being considered as potential drug delivery systems (Gabor and Wirth 2003). The antiproliferative activity of lectins has also been related to their affinity for specific sugar residues exposed on the surface of malignant cells. That is the case of N‐acetyl galactosamine in the Tn antigen that is present in many tumor cells (Fu et al. 2016).

Cancer is one of the most fatal diseases in the world. The plant kingdom represents a promising source of functional components that may be of interest in the treatment of cancer. Extensive glycomic studies have shown that many modifications in the sugars on the surface of cells are associated with malignant cells, and these modifications may in addition facilitate dissemination, metastasis, and invasion of healthy tissues (49). Lectins can identify cancerous cells because they can recognize specific carbohydrates on the cell surface that represent tumor biomarkers (Gurav et al. 2024). Lectins with antiproliferative activity have been isolated from several plants (Konozy and Osman 2022) including genera belonging to *Fabaceae* such as *Astragalus* (Yan et al. 2009), *Bauhinia* (Silva et al. 2014), *Dioclea* (Gondim et al. 2017) and *Phaseolus* (García‐Gasca et al. 2012). These studies found antiproliferative activity against a variety of malignant cellular types, including cancerous cells from breast, lung, colon, liver, and blood (Konozy and Osman 2022).

Our data indicates that the lectin from *V. altissima* inhibits the proliferation of THP‐1 cells, which is a cell line with monocytic characteristics that was originally isolated from the peripheral blood of a one‐yearyear‐old male child with acute monocytic leukemia (Tsuchiya et al. 1980). Other plant lectins have also shown antiproliferative activity against THP‐1 cells, including a lectin purified from banana pulp that exhibited antiproliferative activity at similar concentrations (Batcha et al. 2022). Interestingly, this lectin selectively binds to mannose residues. It has been reported that branched trianntenary mannose glycosides are present in cancer cells. It is possible that the presence of a glycocalyx rich in mannose in THP‐1 cells, as described (Delannoy et al. 2016) is responsible for the binding and antiproliferative activity of the *V. altissima* lectin. A lectin from the *Fabaceae Lotus corniculatus* also showed antiproliferative activity against THP‐1 cells, although this activity was inhibited by galactose and not by mannose or glucose up to 200 mM. Interestingly, this lectin also inhibited the migration of THP‐1 cells, migration being an important step in cancer metastasis. Considering that plant lectins are quite resistant to digestion in the intestinal tract (Vasconcelos and Oliveira 2004), it is possible that peripheral tissues, including blood, might be exposed to lectins if they were absorbed from the gut. Remarkably, plant lectins have been found to be absorbed and even transported to peripheral tissues such as the central nervous system (Zheng et al. 2016).

## 5 Conclusions

A protein exhibiting red blood cell agglutinating activity as well as antiproliferative activity against cancerous cells in vitro has been isolated from the seeds of *V. altissima* , This protein is formed by two identical subunits as determined by native and SDS PAGE. This lectin has potential health‐promoting and diagnostic applications, and it would be of interest to determine whether it inhibits proliferation of cancerous cells other than the THP‐1 cell line. This has found to be the case for other lectins with high affinity for mannose and glucose residues. These potential applications could have a positive effect on the preservation of this wild legume, from which only one population remains in Spain. The subsistence of this endangered population depends on the availability of scarce sources of water in a very arid environment, as reflected by the loss of another population of *V. altissima* , that was present in Spain, due to human intervention resulting in the depletion of aquifers (Lahora et al. 2006). *V. altissima* does not have any legal protection, although it is in the list of endangered “in critical risk” species in the Spanish Vascular Flora (Lahora et al. 2006). The legal protection and *ex situ* conservation in seedbanks of the remaining population in Spain is highly needed.

## Disclosure

The authors have nothing to report.

## Conflicts of Interest

The authors declare no conflicts of interest.

## Data Availability

Data sharing is not applicable to this article as no new data were created or analyzed in this study.
